# Editorial: Dietary nitrate: friend or foe

**DOI:** 10.3389/fnut.2024.1516811

**Published:** 2024-11-26

**Authors:** Liezhou Zhong, Nicola P. Bondonno, Mario Siervo, Catherine P. Bondonno

**Affiliations:** ^1^School of Medical and Health Sciences, Nutrition & Health Innovation Research Institute, Edith Cowan University, Joondalup, WA, Australia; ^2^Institute of Agriculture, The University of Western Australia, Perth, WA, Australia; ^3^Danish Cancer Institute, Copenhagen, Denmark; ^4^School of Population Health, Dementia Centre of Excellence, enAble Institute, Faculty of Health Sciences, Curtin University, Perth, WA, Australia; ^5^Royal Perth Hospital Unit, Medical School, University of Western Australia, Perth, WA, Australia

**Keywords:** nitrate, nitrite, dietary intake, nitrosamine, nitric oxide, health

**Figure 1:**
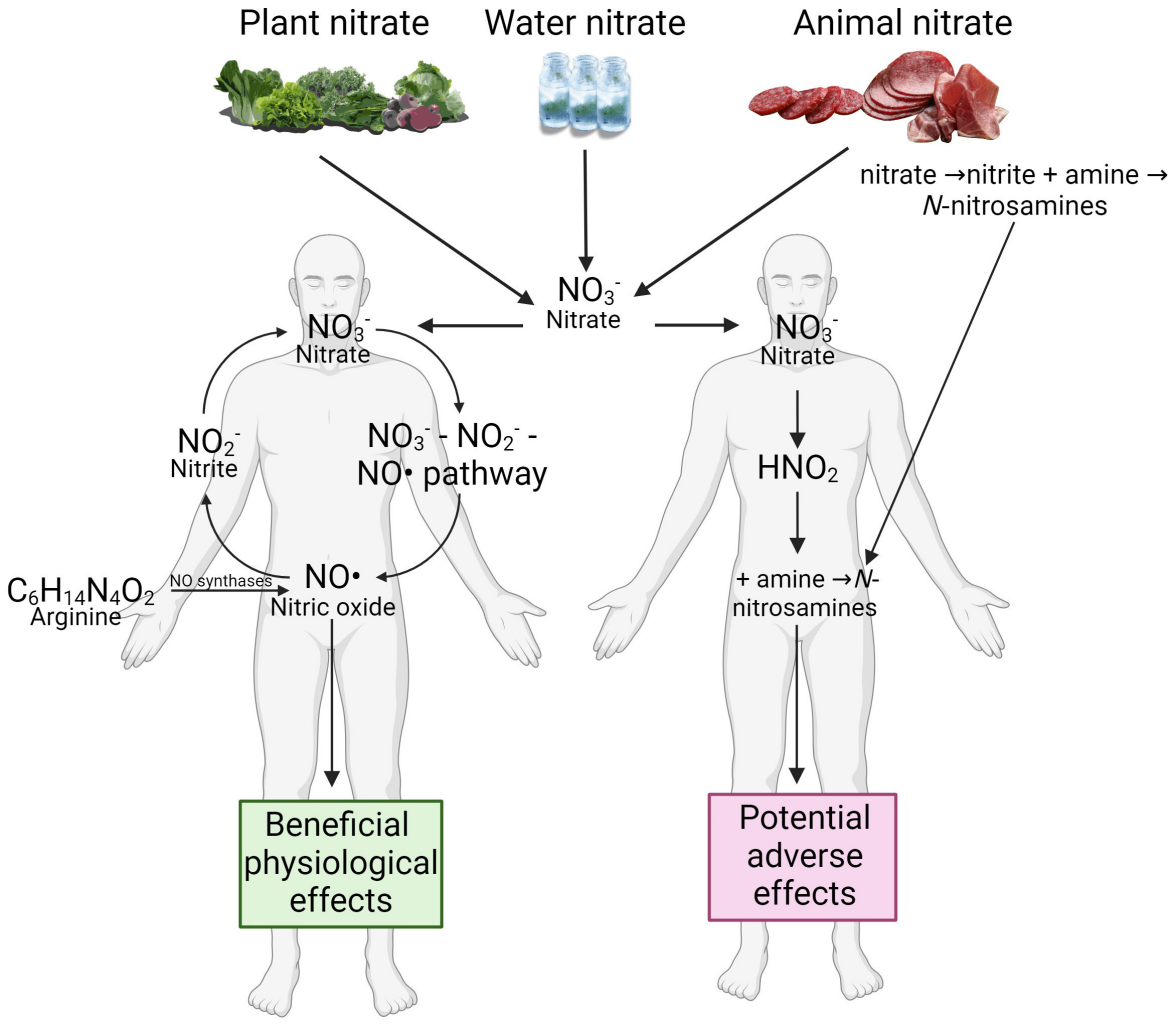
Nitrate dietary source and its health benefits and risks.. Created with BioRender.com.

The health implications of dietary nitrate remain a topic of active debate within the scientific community. The primary sources of dietary nitrate are vegetables (accounting for ~70%−80% of total intake), meat (~10%−15%, where nitrate and nitrite are both naturally occurring and highly regulated added preservatives in processed meat products), and drinking water (~1%−10%, where nitrate is a contaminant) (1–3). The positive health effects of nitrate are primarily mediated by its derivative, nitric oxide (NO), whereas its negative effects may stem from the potential formation of *N*-nitrosamines (2, 4). Nitric oxide plays numerous critical physiological roles, fuelling significant research into the health benefits of dietary nitrate intake. Nitrate (${\text{NO}}_{3}^{-}$) is converted into nitrite (${\text{NO}}_{2}^{-}$), which is then further reduced to NO through an enterosalivary nitrate-nitrite-NO pathway (Figure 1) (5). In contrast, nitrate and nitrite may also contribute to the formation of carcinogenic *N*-nitroso compounds, both exogenously and endogenously (2, 4). The International Agency for Research on Cancer (IARC) has stated that “ingested nitrate or nitrite under conditions that result in endogenous nitrosation is probably carcinogenic to humans (Group 2A).” Recently, the European Commission further restricted the use and maximum levels of nitrate and nitrite as added preservatives in cheeses and processed meats (6). There is now a growing body of evidence, advanced through clinical trials and observational studies, indicating that the health effects of dietary nitrate could depend on its source—vegetables, meat, and water. It is hypothesized that the food matrix and accompanying compounds determine whether dietary nitrate has a health benefit or health risk by influencing whether it leads to the formation of NO or *N*-nitroso compounds (e.g., nitrosamines) (1). The articles in this Research Topic add significant weight in support of this hypothesis.

In a minireview by Zoughaib et al., the authors investigate and discuss the effects of nitrate-rich beetroot juice, now promoted as a supplement for exercise, compared to inorganic nitrate salts on physiological function and exercise capacity. Their conclusion is that beetroot juice outperforms nitrate salts in reducing submaximal oxygen consumption during exercise. The authors hypothesize that the antioxidant and/or anti-inflammatory properties of beetroot, stemming from its rich spectrum of phytonutrients, contribute to these results. However, they caution that it is too soon to draw any firm conclusions and recommend that future studies accurately measure and match the nitrate content in supplements and improve blinding methods by comparing nitrate-containing and nitrate-free versions of beetroot juice and nitrate supplements. They also recommend that studies should focus on reproducible physiological outcomes rather than performance, through the use of involuntary exercise protocols to help mitigate differences in participant expectations and motivations. They raise an important point when considering source—dependent health outcomes of dietary nitrate intake—the presence of other components like potassium and oxalate may limit its use in individuals with health conditions, particularly those with compromised renal function or heart failure.

Comparing nitrate-containing and nitrate-free versions of beetroot juice, Kim et al. and Delgado Spicuzza et al. from the Proctor research lab report results from two randomized, controlled clinical trials. Kim et al. reported that 4–6 days supplementation with nitrate-rich beetroot juice significantly lowered diastolic blood pressure and increased coronary blood flow velocity in individuals with peripheral arterial disease (*n* = 11) at the peak treadmill walking stage. Delgado Spicuzza et al. demonstrated that 7-day nitrate-rich beetroot juice supplementation significantly improved resting endothelial function before ischemia-reperfusion injury in postmenopausal women, compared to nitrate-depleted beetroot juice. While it would be interesting to include nitrate-rich beetroot juice, nitrate-depleted beetroot juice, and nitrate salts in a single trial to compare their health impacts, the results from the current studies highlight the potential role of the food matrix, particularly co-existing compounds, in determining the health functions of dietary nitrate. The reported synergistic effects between plant nitrate and vitamin C (7), and polyphenols (8, 9) are good examples. These reducing compounds can enhance NO production, and mitigate potential adverse effects of dietary nitrate by inhibiting the formation of *N*-nitrosamines (10).

Investigating the “source of nitrate hypothesis” in a large observation study, Erichsen et al. report dietary nitrate intakes in the context of background diet, sociodemographic and lifestyle factors in a Danish cohort of over 55,000 participants. These results are currently being used to inform future studies in this cohort examining source of nitrate and long-term health outcomes. For example, the authors have recently reported that higher plant-sourced nitrate and nitrite intake was associated with a lower risk of all-cause mortality while higher intakes of naturally occurring meat-sourced nitrate and nitrite, additive permitted meat-sourced nitrate and nitrite, and water-sourced nitrate were all linked to higher risks of all-cause mortality, all with potential confounders being adjusted (11). Following the same method of distinguishing between plant- and animal-sourced nitrate, Rajendra et al. reported that, compared to lower intakes, a higher plant-, but not animal-, sourced nitrate intake was associated with a lower risk of dementia-related mortality in Australian Diabetes, Obesity, and Lifestyle (AusDiab) Study (*n* = 9,149). Furthermore, a high, compared to a low, processed meat-sourced nitrate intake was associated with a higher risk of dementia-related mortality. While these studies add weight to the “source of nitrate hypothesis,” more research is required to examine long term health outcomes in the context of higher nitrate intakes and those with impaired nitrate metabolism, the influence of the oral and gut microbiome, as well as factors influencing *N*-nitrosamine formation.

Addressing the nitrate debate is gaining some urgency considering that nitrate-rich plant extracts are being used to replace inorganic nitrate and nitrite salts (E 249–252) in processed meat production in the hope of leveraging the co-existing reducing phytochemicals to inhibit the exogenous and endogenous formation of *N*-nitroso compounds (12). This development has sparked considerable debate related to food labeling and food safety. Simultaneously, a concern has been raised that nitrate-rich beetroot juice supplementation, with or without concurrent vitamin C supplementation, can increase the endogenous formation and excretion of potentially carcinogenic *N*-nitroso compounds (13). Indeed, the health impact of long-term intake of beet-based or other nitrate-rich supplements should be thoroughly explored before promoting these commercial products. This is particularly important given that some supplements that are currently commercialized have extremely high nitrate contents, up to 21,512.4 mg/kg, with some also containing high levels of co-occurring nitrite, up to 1,115.2 mg/kg (14). These values far exceed the regulatory limits for both processed meat products (< 500 mg/kg nitrate, < 200 mg/kg nitrite) and vegetables such as arugula (7,000 mg/kg nitrate, harvested between 1 October and 31 March) which has the highest limit (15). Therefore, consumers should be provided with comprehensive and precise instructions regarding the consumption of these supplements.

The emerging studies surrounding the nitrate debate in this Research Topic, and beyond, are charting the health impact of dietary nitrate and nitrite altering our attitude toward these compounds. However, both the health benefits and potential negative effects point to more sustainable and healthy eating practices: i.e., consuming more vegetables and fewer processed meats. While the nitrate debate is yet to be solved, it is reasonably clear that the food matrix, particularly the co-existing compounds in the wholefood, should be considered. Understanding the complex effects that food structure and co-occurring components have on the nitrate-nitrite-NO pathway from a holistic rather than a nutrient-based perspective, will be critical. The hypothesis that the source of nitrate determines its health effects has yet to be disproven, indeed evidence is rapidly accumulating in its support. This will carry significant weight in shaping international policies governing safe levels of nitrate in both environmental and dietary contexts, currently the acceptable daily intake (ADI) of nitrate, does not differentiate between the sources.
